# Bat cellular immunity varies by year and dietary habit amidst land conversion

**DOI:** 10.1093/conphys/coad102

**Published:** 2024-01-27

**Authors:** Isabella K DeAnglis, Benjamin R Andrews, Lauren R Lock, Kristin E Dyer, Anni Yang, Dmitriy V Volokhov, M Brock Fenton, Nancy B Simmons, Cynthia J Downs, Daniel J Becker

**Affiliations:** Department of Environmental Biology, SUNY College of Environmental Science and Forestry, 1 Forestry Drive, Syracuse, NY, 13210, USA; Department of Biological Sciences, University of Arkansas, 1 University of Arkansas, Fayetteville, AR, 72701, USA; Department of Environmental Biology, SUNY College of Environmental Science and Forestry, 1 Forestry Drive, Syracuse, NY, 13210, USA; School of Biological Sciences, University of Oklahoma, 730 Van Vleet Oval, Norman, OK, 73019, USA; School of Biological Sciences, University of Oklahoma, 730 Van Vleet Oval, Norman, OK, 73019, USA; Department of Geography and Environmental Sustainability, University of Oklahoma, 100 East Boyd St, Norman, OK, 73019, USA; Center for Biologics Evaluation and Research, Food and Drug Administration, 10903 New Hampshire Avenue, Silver Spring, MD, 20993, USA; Department of Biology, University of Western Ontario, 1151 Richmond Street, London, Ontario, N6A 3K7, Canada; Department of Mammalogy, Division of Vertebrate Zoology, American Museum of Natural History, 200 Central Park West, New York, NY, 10024, USA; Department of Environmental Biology, SUNY College of Environmental Science and Forestry, 1 Forestry Drive, Syracuse, NY, 13210, USA; School of Biological Sciences, University of Oklahoma, 730 Van Vleet Oval, Norman, OK, 73019, USA

**Keywords:** bacterial pathogens, cellular immunity, Chiroptera, ecoimmunology, land conversion

## Abstract

Monitoring the health of wildlife populations is essential in the face of increased agricultural expansion and forest fragmentation. Loss of habitat and habitat degradation can negatively affect an animal’s physiological state, possibly resulting in immunosuppression and increased morbidity or mortality. We sought to determine how land conversion may differentially impact cellular immunity and infection risk in Neotropical bats species regularly infected with bloodborne pathogens, and to evaluate how effects may vary over time and by dietary habit. We studied common vampire bats (*Desmodus rotundus*), northern yellow-shouldered bats (*Sturnira parvidens*) and Mesoamerican mustached bats (*Pteronotus mesoamericanus*), representing the dietary habits of sanguivory, frugivory and insectivory respectively, in northern Belize. We compared estimated total white blood cell count, leukocyte differentials, neutrophil to lymphocyte ratio and infection status with two bloodborne bacterial pathogens (*Bartonella* spp. and hemoplasmas) of 118 bats captured in a broadleaf, secondary forest over three years (2017–2019). During this period, tree cover decreased by 14.5% while rangeland expanded by 14.3%, indicating increasing habitat loss and fragmentation. We found evidence for bat species-specific responses of cellular immunity between years, with neutrophil counts significantly decreasing in *S. parvidens* from 2017 to 2018, but marginally increasing in *D. rotundus*. However, the odds of infection with *Bartonella* spp. and hemoplasmas between 2017 and 2019 did not differ between bat species, contrary to our prediction that pathogen prevalence may increase with land conversion. We conclude that each bat species invested differently in cellular immunity in ways that changed over years of increasing habitat loss and fragmentation. We recommend further research on the interactions between land conversion, immunity and infection across dietary habits of Neotropical bats for informed management and conservation.

## Teaser

Understanding how land conversion affects immunity against pathogens in wildlife can inform conservation decisions and our understanding of pathogen dynamics in host communities. We found evidence that Neotropical bats invested differently in cellular immunity over years of land conversion, suggesting that investment in immune defenses varies by species and diet.

## Introduction

Agriculture expansion fragments and degrades landscapes, reducing suitable habitat available to many terrestrial mammals (Laurance *et al.,* 2014; Crooks *et al.,* 2017). Tropical areas worldwide are experiencing rapid human population growth and associated agriculture expansion, which is particularly concerning for conservation since the tropics support about two thirds of global biodiversity (Bradshaw *et al.,* 2009; Antonelli, 2022). Impacts of land conversion on animal physiology, and ultimately on fitness, depend on phenotypic plasticity and how habitat loss and fragmentation affect specific aspects of an animal’s ecology. Generalists may be less likely to be affected by land conversion than specialists (Ramiadantsoa *et al.,* 2018), and some species may be epigenetically primed to take advantage of changing landscapes (Kilvitis *et al.,* 2017). Species with specialized niches, including highly specific shelter or dietary needs, are more likely to be affected by the physiological challenges associated with habitat loss and fragmentation, resulting in population declines in such species (Kosydar *et al.,* 2014). Conversely, some species may be less affected by or even benefit from land conversion because of increased access to human-provisioned food resources in the form of crops or livestock (Oro *et al.,* 2013). The dietary habit of a species can thus impact how habitat changes associated with land conversion affect individual physiology (Hinam and Clair, 2008). Reduced physiological condition, as a result of habitat loss and fragmentation, can result in immunodeficiency and increased morbidity (Villafuerte *et al.,* 1997; Johnstone *et al.,* 2012; Seltmann *et al.,* 2017). Understanding how land conversion differentially affects the ability of species to mount immune defenses against pathogens can inform conservation and land management decisions and contribute significantly to our understanding of pathogen dynamics in complex host communities.

Land use changes have been linked to changes in immune phenotype and glucocorticoid concentration in birds and mammals (Messina *et al.,* 2018). In some species, land conversion may cause immunosuppression and immunomodulation, resulting in increased susceptibility to infections in wildlife (Aguirre and Tabor, 2008; Belasen *et al.,* 2019). When an individual is experiencing more frequent or intense stressors (Davis *et al.,* 2008) or is parasitized (Hernandez *et al.,* 2018), plasma glucocorticoid levels can increase, often impairing the host immune response (Sapolsky *et al.,* 2000; Coutinho and Chapman, 2011). Comparing estimated total white blood cell counts (TWBC) and differential white blood cell counts (DWBC) in mammals under different environmental conditions (i.e. in areas with differential levels of land conversion) is a low-cost and tractable means for determining how environmental changes impact individual investment in cellular immune defenses (Schneeberger *et al.,* 2013; Becker *et al.,* 2018*b*). For example, increased glucocorticoid levels are associated with increased TWBC, elevated neutrophil to lymphocyte ratio (NL ratio) and eosinopenia across vertebrates (Jain, 1986; Davis *et al.,* 2008). Further, immunocompromised hosts often experience increased susceptibility to infection, and acute bacterial infection in particular can increase TWBC counts and cause neutrophilia, lymphopenia and monocytosis in vertebrates (Jain, 1986; Davis *et al.,* 2004; Davis *et al.,* 2008). Thus, documenting changes in the leukocyte profile and infection status of wildlife over time can help evaluate how land conversion impacts host cellular immunity and pathogen spread.

The Neotropics contain the greatest diversity of bats globally, with species that span almost all possible mammal dietary habits (Fenton, 1992; Mickleburgh *et al.,* 2002). Neotropical bats are important members of tropical forest ecosystems because of their role in seed dispersal, insect predation and pollination (Kunz *et al.,* 2011). Bats are also notable for their potential to spread virulent pathogens to humans, livestock and other species (Chan *et al.,* 2013; Allocati *et al.,* 2016). Humans that live in or near fragmented forests in the tropics have greater exposure to bat-borne pathogens due to their proximity to bat hosts at ecotone roost sites (Rulli *et al.,* 2017). Because of their great ecological diversity, Neotropical bats are a valuable system for studying effects of land conversion on diverse ecological interactions.

In this study, we used Neotropical bats as a system to ask how decreasing forest cover and increasing rangeland resulting from conversion affect cellular immunity and infection status of diverse host species, with the goal of testing how dietary habit impacts the immune response to land conversion. Specifically, we studied common vampire bats (*Desmodus rotundus*), northern yellow-shouldered bats (*Sturnira parvidens*) and Mesoamerican mustached bats (*Pteronotus mesoamericanus*). These species represent the dietary habits of sanguivores, frugivores and insectivores, respectively (Bobrowiec *et al.,* 2015; Ingala *et al.,* 2021). All three bats are broadly distributed in Central America and are routinely found in landscapes affected by land conversion (Kraker-Castañeda *et al.,* 2016; Herrera *et al.,* 2018; Alpízar *et al.,* 2019; Brändel *et al.,* 2020); however, their response to forest loss and fragmentation likely differs owing to their different dietary habits. Across its range, *Desmodus rotundus* capitalizes on domestic animal prey (especially cattle) in rangeland dominated landscapes, and this human-provisioned food source is preferentially selected over wildlife prey (Voigt and Kelm, 2006; Bobrowiec *et al.,* 2015; Ingala *et al.,* 2019). Therefore, we predict that the cellular immunity of *D. rotundus* would not be negatively impacted by land conversion (or even could benefit from livestock prey; Becker *et al.,* 2018*b*). *Sturnira parvidens* is a frugivore that specializes in fruit from early successional plants, which are most abundant in fragmented and disturbed forests (Galindo-Gonzalez *et al.,* 2000; García-Morales *et al.,* 2012; Kraker-Castañeda *et al.,* 2016). Prior work has found that *S. parvidens* is one of the most abundant bat species in at least some fragmented forests (Ramírez-Lucho *et al.,* 2017; Herrera *et al.,* 2018). In contrast, *Pteronotus mesoamericanus* may be more vulnerable to increasing habitat loss and fragmentation, as these bats forage for insects using echolocation in dense vegetation within forest interiors (Alpízar *et al.,* 2019; Núñez *et al.,* 2019). Fragmentation resulting from land conversion could thus reduce access to forest interiors for bat foraging (Núñez *et al.,* 2019), which could function as a stressor that impairs *P. mesoamericanus* immunity.

The likelihood of a bat being exposed to pathogens and the mode of pathogen exposure can also depend on the ecological niche of a species (Schneeberger *et al.,* 2013). In this study, we focused on how land conversion affects the prevalence of *Bartonella* spp. and hemotropic *Mycoplasma* spp. (i.e. hemoplasmas), which are common bacterial pathogens in Neotropical bats (Ikeda *et al.,* 2017; Becker *et al.,* 2018*a*; Becker *et al.,* 2018*b*; Becker *et al.,* 2020; Volokhov *et al.,* 2023). *Bartonella* spp. are intraerythrocytic and are vectored by hematophagous arthropods, including bat ectoparasites, and may also be transmitted through blood, saliva, or feces to a variety of hosts (Jacomo *et al.,* 2002). In humans, *Bartonella* spp. can cause a variety of diseases, including cat-scratch disease, endocarditis and Carrion's disease (Jacomo *et al.,* 2002). Hemoplasmas are parasites of erythrocytes and are thought to be transmitted by direct contact and possibly also vectored by hematophagous arthropods, including bat ectoparasites (Messick, 2004; Cohen *et al.,* 2018). Hemoplasmas can cause hemolytic anemia, arthritis, pneumonia, conjunctivitis, infertility, and other acute to chronic diseases in humans and other mammals (Messick, 2004; Descloux *et al.,* 2021; Millán *et al.,* 2021). Due to the potential of these pathogens to cause zoonotic infections, which can be life-threatening in humans, understanding how the likelihoods of infection with *Bartonella* spp. and hemoplasmas in bats are affected by land conversion is important to forecast or prevent future pathogen spillover. Here, we compared the leukocyte profiles and infection status of *D. rotundus, S. parvidens* and *P. mesoamericanus* inhabiting northern Belize over a three-year period of decreasing forest cover. We hypothesized that increased habitat loss and fragmentation would impact the species’ cellular immunity and infection status differentially, according to their dietary habit, with *P. mesoamericanus* experiencing the greatest changes in cellular immunity (i.e. increased TWBC counts, monocytosis, neutrophilia and lymphopenia) and infection status (i.e. increased odds of infection) and *D. rotundus* experiencing only minor changes in cellular immunity and infection status, due to their foraging ecology. Evaluating the immunological impacts of land conversion on these bat species is important not only to inform bat conservation efforts, but also to predict how future habitat loss can influence pathogen spread in bats and potentially to humans.

## Materials and Methods

### Bat capture and sampling

As part of broader ecological, immunological and epidemiological studies of bats in Belize (Herrera *et al.,* 2018; Becker *et al.,* 2020; Becker *et al.,* 2021*a*; Becker *et al.,* 2022), we sampled *D. rotundus*, *S. parvidens* and *P. mesoamericanus* during April to May 2017–2019 within the Lamanai Archaeological Reserve (LAR) of Orange Walk District, Belize (N 17.76343, W 88.65292). The LAR is a broadleaf, secondary protected forest near the New River Lagoon, for which the surrounding matrix is experiencing increasing forest loss and fragmentation as land is converted to cropland and cattle pastures (Herrera *et al.,* 2018; Ingala *et al.,* 2019, Figure 1). As described previously, we used mist nets and harp traps to capture bats along flight paths and occasionally at the exits of roosts from 19:00 hours until 22:00 hours (Becker *et al.,* 2020; Becker *et al.,* 2021*b*). Bats were kept in clean cloth bags prior to processing and were identified and sexed based on morphology (e.g. Reid, 1997). Between 3 and 30 μl of blood were sampled, based on bat body mass, by lancing the propatagial vein with a sterile needle (23–30G) and collected in a heparinized capillary tube. Thin blood smears were prepared on glass slides and stained with Wright–Giemsa (Astral Diagnostics Inc., Hematology Stain Set Quick III), and remaining blood was stored on Whatman FTA cards at room temperature. All bats for this study were released following sampling. Field procedures were performed according to guidelines for the safe and humane handling of bats published by of the American Society of Mammalogists (Sikes *et al.,* 2016) and were approved by the Institutional Animal Care and Use Committees of the University of Georgia (A2014 04-016-Y3-A5) and American Museum of Natural History (AMNHIACUC-20170403, AMNHIACUC-20180123, AMNHIACUC-20190129). Fieldwork and sampling were authorized by the Belize Forest Department under permits WL/2/1/17(16), WL/2/1/17(19), WL/2/1/18(16) and FD/WL/1/19(09).

**Figure 1:**
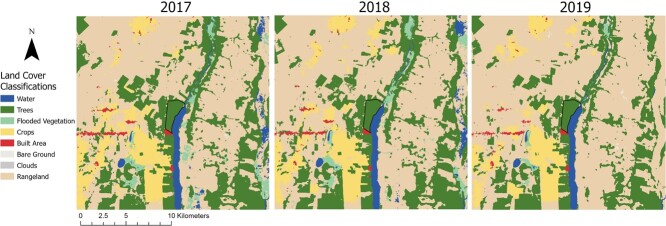
Map of land cover change in northern Belize, within 10 km of the LAR, from 2017 to 2019. The 450-ha forest of the LAR is shown in black (Becker *et al.,* 2020). Map and land use/land cover data were derived from the Sentinel-2 10 m land use/land cover time series of the world produced by Impact Observatory, Microsoft and Esri (Karra *et al.,* 2021). This dataset is based on the data produced for the Dynamic World Project by National Geographic Society in partnership with Google and the World Resources Institute.

### Land cover change quantification

Land cover data were derived from the Sentinel-2 10-m land use/land cover time series of the world produced by Impact Observatory, Microsoft and Esri (Karra *et al.,* 2021) and imported into ArcGIS Pro (ESRI Inc., Redland, CA). Using the LAR as a central point, the resulting map was cropped to a 10 km extent, representing a conservative boundary of bat movement based on past estimates of Neotropical bat home ranges, including in this Belize study site (Fleming *et al.,* 1972; Trajano, 1996; Fenton *et al.,* 2000; Becker *et al.,* 2021*b*). After masking the LAR, individual binary layers were created for each land cover type in the matrix on May 1 of each year of the study (2017, 2018 and 2019), where a value of 1 indicated pixels mapping to a single land cover classification and a value of 0 indicated pixels mapping to all other land cover classifications. As Sentinel-2 collects data every five days, May 1 was selected because it approximately marks the midpoint sampling period and because cloud-cover was minimal during each year. Total pixel counts for each land cover classification per year were plotted using the *ggplot2* package in R (Wickham, 2016). The net change for each land cover classification was quantified by subtracting the 2019 binary layer from the 2017 binary layer. The number of pixels with a value of +1 and the number of pixels with a value of −1 in the resulting layer were each divided by the total number of pixels mapped to that land cover classification in 2017 to determine the percent gained and lost, respectively. The percent lost was subtracted by the percent gained to determine the net percent change.

### White blood cell counts and statistical analyses

We estimated TWBC counts for each blood smear (*n* = 118) by averaging the number of leukocytes (neutrophils, lymphocytes, monocytes, eosinophils, basophils) under 10 random fields under 400X magnification (Schneeberger *et al.,* 2013). DWBC counts were then estimated by identifying 100 leukocytes and recording the relative abundance of each type of white blood cell under 1000× (oil immersion) magnification. The absolute number of each white blood cell type was then determined by multiplying its relative abundance by the estimated TWBC count (Becker *et al.,* 2021*a*), and we derived NL ratios. A subset of the hematology data from 2017 and 2018 were published previously (Cornelius Ruhs *et al.,* 2021; Becker *et al.,* 2021*a*).

We used generalized linear models (GLMs) to test how each of our cellular immunity measures (TWBC, absolute neutrophils, absolute lymphocytes, NL ratios, absolute monocytes, absolute eosinophils, and absolute basophils) varied among our three species across the three years. We fit separate GLMs with immunity predicted by year, bat species and their interaction (see Becker *et al.,* 2020 for sex effects and other individual-level covariates), modeling each response with a Tweedie distribution (Dunn and Smyth, 2005). We used the *mgcv* package in R to fit Tweedie-distributed GLMs using maximum likelihood (Wood, 2006). We adjusted for the inflated false-discovery rate in *post-hoc* comparisons with the *emmeans* package (Benjamini and Hochberg, 1995).

### *Bartonella* spp. *and hemoplasma infection analyses*

We expanded prior surveys of *Bartonella* spp. and hemoplasmas in Belize with analyses of paired blood samples from 2019 bats with blood smears (Volokhov *et al.,* 2017; Becker *et al.,* 2018*a*; Becker *et al.,* 2020; Becker *et al.,* 2021*a*; Volokhov *et al.,* 2023). We extracted DNA from Whatman FTA cards using Qiagen QIAamp DNA Investigator Kits (Volokhov *et al.,* 2017). We then used PCR and gel electrophoresis to determine the presence of *Bartonella* spp. (targeting the *gltA* gene) and hemoplasmas (targeting the 16S rRNA gene) with primers and procedures described previously (Volokhov *et al.,* 2017; Becker *et al.,* 2018*a*; Becker *et al.,* 2020). The amplicons were directly sequenced by Sanger method and sequence analysis was performed for hemoplasmas to assess similarity to our previously established genotypes in Belize bats (see Supplemental Information; Becker *et al.,* 2020).

Following our analyses of white blood cell data, we derived infection prevalence and 95% confidence intervals (Wilson interval) for each pathogen (and for co-infection) using the *prevalence* package. We then fit GLMs with a binomial distribution per pathogen, with infection status predicted by year, bat species and their interaction (see Becker *et al.,* 2020 for effects of sex and other individual-level covariates on a larger sample size of Belize bat infection status). Because of low sample sizes for pathogen analyses in 2018, we limited this comparison to 2017 and 2019 (*n* = 89). To account for overall smaller sample sizes here, we used the *brglm* package to implement Firth’s bias reduction (Firth, 1993).

## Results

### Land cover change

**Figure 2:**
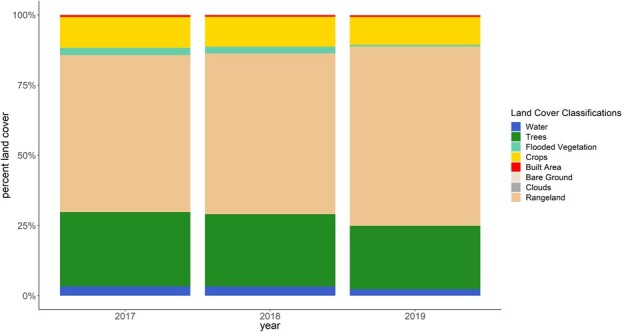
Change in land cover in the habitat matrix within 10 km of the LAR from 2017 to 2019.

The habitat matrix within 10 km of our study site was dominated by rangeland during every year of bat sampling, followed by trees and crops. Land cover change across years was characterized by an expansion of rangeland that coincided with a reduction of all other land cover classifications except for bare ground. Notably, tree cover was reduced by 14.5% from 2017 to 2019 while rangeland expanded by 14.3% (Figure 2).

**Table 1:** Results of Tweedie-distributed GLMs predicting each cellular immunity measure by bat species, year and their interaction

Response	Predictor	*F*	*p*
Total leukocytes *R^2^* = 0.05	species	0.86	0.43
	year	1.78	0.17
	species^*^year	**3.91**	**<0.01**
Absolute neutrophils *R^2^* = 0.33	species	1.13	0.33
	year	2.15	0.12
	species^*^year	**3.34**	**0.01**
Absolute lymphocytes *R^2^* = 0.32	species	2.55	0.08
	year	2.31	0.10
	species^*^year	2.04	0.09
NL ratios *R^2^* = 0.08	**species**	**5.38**	**<0.01**
	year	0.18	0.84
	species^*^year	1.16	0.33
Absolute monocytes *R^2^* = 0.05	**species**	**4.95**	**<0.01**
	**year**	**4.26**	**0.02**
	species^*^year	**2.78**	**0.03**
Absolute eosinophils *R^2^* = 0.15	**species**	**5.18**	**<0.01**
	**year**	**4.29**	**0.02**
	species^*^year	**2.81**	**0.03**
Absolute basophils *R^2^* = 0.06	species	2.37	0.10
	**year**	**3.06**	**0.05**
	species^*^year	**2.63**	**0.04**

### Cellular immunity

**Figure 3:**
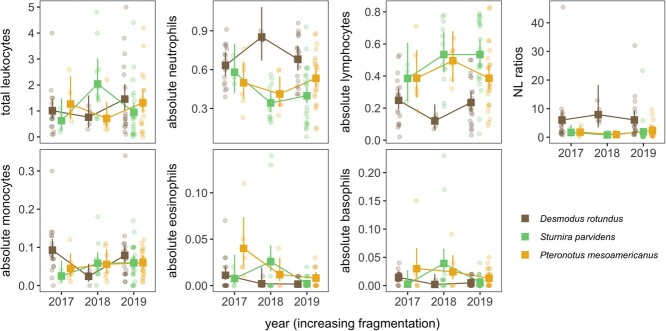
Predicted means and 95% confidence intervals from Tweedie GLMs with interactive effects of bat species and year, for each cellular immunity measure. Raw data are overlaid and jittered.

We estimated TWBC and DWBC counts from 42 *D. rotundus*, 40 *S. parvidens* and 36 *P. mesoamericanus*, in the LAR between 2017 and 2019. Our GLMs found generally strong support for species-specific responses of cellular immunity to year (species-by-year interaction: *F_4_* = 1.16–3.91, *p* = 0.01–0.33; Table 1, Figure 3). For total leukocytes, the predicted means suggested TWBC of *S. parvidens* increased between 2017 and 2018 and decreased from 2018 to 2019 whereas TWBC of other species did not change over time, but these *S. parvidens* contrasts were not significant after adjusting for multiple comparisons (Supplementary Table S1 and Table 2). In contrast, although absolute neutrophil counts did not differ between species in 2017, temporal patterns varied among bats in subsequent years. Absolute neutrophil counts of *S. parvidens* declined from 2017 to 2018, whereas absolute neutrophil count marginally increased in *D. rotundus* between these same years (Supplementary Table S2 and Table 2). Absolute lymphocyte counts showed a weaker interactive effect of bat species and year (*F_4_* = 2.04, *p* = 0.09), with *S. parvidens* and *P. mesoamericanus* generally having 1.8 and 2.3 times as many lymphocytes as *D. rotundus* (Supplementary Table S3 and Table 2). NL ratios did not vary by year (*F_4_* = 1.16, *p* = 0.33), but both *S. parvidens* and *P. mesoamericanus* had lower NL ratios than *D. rotundus* (Supplementary Table S4 and Table 1). For absolute monocyte counts, predicted means suggested temporal variability in *D. rotundus* and little change for *S. parvidens* or *P. mesoamericanus*, but these contrasts were likewise not significant after adjusting for multiple comparisons (Supplementary Table S5 and Table 2). Absolute eosinophil counts demonstrated a significant species-by-year interaction, where these cells decreased from 2018 to 2019 in *S. parvidens* and marginally decreased in *P. mesoamericanus* between 2017 and 2018 (Supplementary Table S6 and Table 2). For absolute basophil counts, *S. parvidens* showed a marginal increase from 2017 to 2018 and a significant decrease from 2018 to 2019, while absolute basophil counts of other species did not change over time (Supplementary Table S7 and Table 2).

### Bloodborne pathogen infections

**Figure 4:**
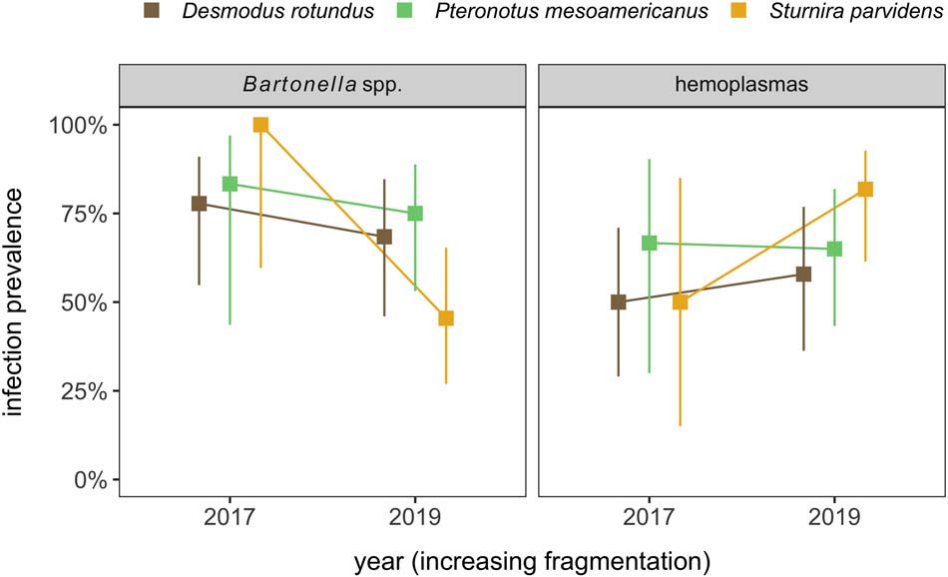
Prevalence of *Bartonella* spp. and hemoplasma infection alongside 95% confidence intervals (Wilson’s interval) stratified by bat species and year.

For the 89 bats screened for bacterial infections in 2017 and 2019 (37 *D. rotundus*, 28 *S. parvidens*, 29 *P. mesoamericanus*), 69% were positive for *Bartonella* spp. (CI: 58–77%), 64% were positive for hemoplasmas (CI: 54–73%) and 45% had coinfections (CI: 35–55%). Our GLMs revealed no effects of species, year, or their interaction on the probability of infection for either *Bartonella* spp. or hemoplasmas (Table 3). Such results thereby suggest little shift in these potentially chronic infections over time on a per-species basis (Figure 4), although such conclusions may be limited by the smaller sample sizes here.

Sequencing of hemoplasma positives from 2019 updated a long-term dataset of the diversity of these pathogens in our study area (Volokhov *et al.,* 2017; Becker *et al.,* 2020; Volokhov *et al.,* 2023; see Supplemental Materials for more information). We confirmed previously established genotypes in each of our three species (*D. rotundus*: VBG1, VBG2, VBG3; *S. parvidens*: SP1 groups A–C; *P. mesoamericanus*: PPM1). One *S. parvidens* had a novel genotype (SP2, GenBank accession OQ308927) showing 97% similarity to the APH3 genotype, observed only in *Artibeus intermedius* (Becker *et al.,* 2020). *P. mesoamericanus* was also infected with the SP1 genotype and with new *Pteronotus*-specific genotype (PPM2, GenBank accession OQ308895; 97% similar to VBG1). Lastly, *P. mesoamericanus* was also infected by a novel non-hemotropic *Mycoplasma*, the *M. moatsii*–like genotype 4 (GenBank accession OQ308889). All 16S rRNA sequence data are available on GenBank through accession OQ308885-7, OQ308889, OQ308893, OQ308895-97, OQ308899-900, OQ308902-12, OQ308914-19, OQ308923-24, OQ308926-27, OQ533048, OQ546518-19, OQ546527-28, OQ546533, OQ546547, OQ546552, and OQ546555.

## Discussion

By examining changes in leukocyte profiles and bloodborne pathogen (*Bartonella* spp. and hemoplasmas) prevalence in bat species representing distinct dietary habits over years of increasing habitat loss and fragmentation, we expanded our understanding of how anthropogenic habitat modification may differentially affect immune phenotype and infection prevalence of bats. Between 2017 and 2019, during a period of land conversion characterized by a reduction in tree cover and an expansion of rangeland, *D. rotundus, S. parvidens* and *P. mesoamericanus* exhibited differences in cellular immune defenses. These findings provide initial support for our prediction that hosts belonging to distinct dietary habits are differentially affected by a changing landscape over time and that these impacts manifest in changes to immune strategy. However, within this sample of bats, we found that *Bartonella* spp. and hemoplasma infection risk did not deviate among years or species, which is consistent with the findings that there were no significant changes in hemoplasma infection prevalence from 2017 to 2018 in *D. rotundus* (Becker *et al.,* 2020). Interestingly, temporal and dietary differences in bat cellular immunity did not translate into variation in *Bartonella* spp*.* nor hemoplasma risk, at least not over the time scale of this study.

Hosts invest differently in their immune systems based on the varied costs associated with innate and adaptive immunity (Chaplin, 2010). Although investing more energy into adaptive immunity may be more energetically costly during development, innate immunity can also be costly to maintain later in life when an individual acquires a pathogen (Klasing, 2004; McDade *et al.,* 2016). Among other factors, life history traits (Lochmiller and Deerenberg, 2000), lifespan (Previtali *et al.,* 2012) and resource availability (Becker *et al.,* 2018*b*) can influence immune investment. Thus, it is likely that species of bats with different ecological niches will show distinct immune investment strategies. For example, a study of the bat community at our study site previously found species-specific differences in the relationship between mercury and cellular immunity, wherein bats that rely on aquatic prey and bats in agricultural habitats had higher mercury levels and neutropenia as compared to bats of other ecological niches (Becker *et al.,* 2021*a*). Therefore, the distinct ecological requirements of *D. rotundus, S. parvidens* and *P. mesoamericanus* may drive species-level variation in how these bats invest in immunity (and in turn how this is affected by land conversion over time).

Temporal patterns of immune investment, as measured by leukocyte profiles, varied among bat species, which could at least partially arise from differences in dietary habits. Because diet will likely impact the probability of acquiring a pathogen (Han *et al.,* 2021) and can also directly shape immune phenotypes (Schneeberger *et al.,* 2013), bat dietary habits will likely affect immune changes over time. Differences in dietary habits may explain why neutrophil counts declined in *S. parvidens* between 2017 and 2018, while neutrophil counts marginally increased in *D. rotundus*. If *S. parvidens* encounters fewer pathogens when feeding on contaminated fruit, it may be energetically favorable for them to invest less in innate immunity (i.e. decreased neutrophil count). Conversely, if *D. rotundus* encounters more pathogens when feeding on infected blood, it may be beneficial for them to increase absolute neutrophil counts to avoid mounting an energetically costly immune response during periods of increased stress, such as what might be caused by habitat degradation, roost disturbance or forest fragmentation (Read and Allen, 2000; Allen *et al.,* 2008). However, NL ratios did not vary among years for any of the species, suggesting that it is unlikely that increased glucocorticoid levels play a major role in changing leukocyte counts over time. Similarly, dietary habits may also explain why eosinophil counts decreased in *S. parvidens* from 2018 to 2019, but did not change significantly in *D. rotundus* or *P. mesoamericanus,* in the event that frugivorous feeding behaviours are linked to ingesting the eggs or larvae of helminths, leading to variable helminth infections (which are often characterized by increased eosinophil counts; Huang and Appleton, 2016).

**Table 2:** Post-hoc comparisons among years within each bat species for each cellular immunity measure. Results are subset from the full pairwise post-hoc analyses following adjustment for multiple comparisons (see Supplementary Tables S1–S7 for the complete list of comparisons)

**Response**	**Contrast**	**Ratio**	**SE**	***t***	***p***
Total leukocytes	*D. rotundus* 2017/2018	1.33	0.56	0.68	0.67
	*D. rotundus* 2018/2019	0.52	0.22	−1.57	0.36
	*S. parvidens* 2017/2018	0.31	0.15	−2.45	0.14
	*S. parvidens* 2018/2019	2.15	0.56	2.94	0.12
	*P. mesoamericanus* 2017/2018	1.77	0.80	1.27	0.39
	*P. mesoamericanus* 2018/2019	0.54	0.20	−1.7	0.33
Absolute neutrophils	*D. rotundus* 2017/2018	0.74	0.11	−2.07	0.09
	*D. rotundus* 2018/2019	1.25	0.18	1.60	0.18
	***S. parvidens* 2017/2018**	**1.69**	**0.34**	**2.60**	**0.03**
	*S. parvidens* 2018/2019	0.86	0.12	−1.06	0.39
	*P. mesoamericanus* 2017/2018	1.21	0.25	0.93	0.44
	*P. mesoamericanus* 2018/2019	0.77	0.12	−1.60	0.18
Absolute lymphocytes	*D. rotundus* 2017/2018	2.07	0.71	2.13	0.07
	*D. rotundus* 2018/2019	0.51	0.17	−1.96	0.10
	*S. parvidens* 2017/2018	0.72	0.19	−1.25	0.28
	*S. parvidens* 2018/2019	1.00	0.15	0.00	1.00
	*P. mesoamericanus* 2017/2018	0.78	0.19	−1.00	0.41
	*P. mesoamericanus* 2018/2019	1.28	0.24	1.33	0.26
NL ratios	*D. rotundus* 2017/2018	2.07	0.71	2.13	0.07
	*D. rotundus* 2018/2019	0.51	0.17	−1.96	0.10
	*S. parvidens* 2017/2018	0.72	0.19	−1.25	0.28
	*S. parvidens* 2018/2019	1.00	0.15	0.00	1.00
	*P. mesoamericanus* 2017/2018	0.72	0.16	−1.44	0.23
	*P. mesoamericanus* 2018/2019	1.28	0.24	1.33	0.26
Absolute monocytes	*D. rotundus* 2017/2018	3.87	1.79	2.91	0.14
	*D. rotundus* 2018/2019	0.30	0.14	−2.56	0.14
	*S. parvidens* 2017/2018	0.43	0.23	−1.60	0.24
	*S. parvidens* 2018/2019	0.98	0.25	−0.08	0.98
	*P. mesoamericanus* 2017/2018	0.81	0.34	−0.50	0.77
	*P. mesoamericanus* 2018/2019	0.93	0.29	−0.23	0.96
Absolute eosinophils	*D. rotundus* 2017/2018	5.56	6.68	1.43	0.25
	*D. rotundus* 2018/2019	1.27	1.69	0.18	0.94
	*S. parvidens* 2017/2018	0.29	0.23	−1.54	0.23
	***S. parvidens* 2018/2019**	**12.4**	**7.25**	**4.31**	**<0.01**
	*P. mesoamericanus* 2017/2018	3.37	1.91	2.14	0.08
	*P. mesoamericanus* 2018/2019	1.47	0.83	0.69	0.61

(Continued)

**Table 2:** Continued

**Response**	**Contrast**	**Ratio**	**SE**	***t***	***p***
Absolute basophils	*D. rotundus* 2017/2018	7.22	8.77	1.63	0.19
	*D. rotundus* 2018/2019	0.42	0.53	−0.69	0.61
	*S. parvidens* 2017/2018	0.06	0.08	−2.22	0.10
	***S. parvidens* 2018/2019**	**6.71**	**3.01**	**4.24**	**<0.01**
	*P. mesoamericanus* 2017/2018	1.23	0.70	0.36	0.76
	*P. mesoamericanus* 2018/2019	1.87	0.92	1.27	0.30

**Table 3:** Results of binomial GLMs predicting *Bartonella* spp. or hemoplasma infection status by bat species, year and their interaction. Reference levels are *D. rotundus* for bat species and 2017 for year

Response	Coefficient	OR	*z*	*p*
*Bartonella* spp. *R^2^* = 0.09	intercept	3.22	2.11	0.03
	*P. mesoamericanus*	1.14	0.11	0.91
	*S. parvidens*	2.79	0.58	0.56
	2019	0.64	−0.59	0.55
	*P. mesoamericanus*: 2019	1.19	0.13	0.90
	*S. parvidens:* 2019	0.14	−1.03	0.30
Hemoplasmas *R^2^* = 0.06	intercept	1.00	0.00	1.00
	*P. mesoamericanus*	1.80	0.60	0.55
	*S. parvidens*	1.00	0.00	1.00
	2019	1.35	0.46	0.65
	*P. mesoamericanus:* 2019	0.74	−0.26	0.80
	*S. parvidens:* 2019	3.03	0.85	0.40

Other factors that may influence patterns observed in bat leukocyte profiles among species include sociality and roosting behaviour (Calisher *et al.,* 2006; Mühldorfer *et al.,* 2011). Both *D. rotundus* and *P. mesoamericanus* are highly social species that live in colonies of a few thousand individuals (Wilkinson, 1985; Wilkinson, 1986; Santana *et al.,* 2011; Clement and Kanwal, 2012; Becker *et al.,* 2020), while *S. parvidens* roost alone or in groups of up to 10 individuals (Fenton *et al.,* 2000; Evelyn and Stiles, 2003). Highly social species, such as *D. rotundus* and *P. mesoamericanus*, may experience greater pathogen risk compared to less social species, such as *S. parvidens* (Stanko *et al.,* 2002; Webber and Willis, 2016). Moreover, roosts that are permanent and protected from precipitation, such as caves, have increased infection risk compared to more ephemeral or less protected roosts, such as tree cavities (Patterson *et al.,* 2007). Therefore, species, such as *D. rotundus* and *P. mesoamericanus,* which roost more commonly in caves, could have increased infection risk compared to bats that only inhabit caves rarely and only at night for brief periods (e.g. *S. parvidens*; Sapey, 2019). Variation in infection risk because of sociality, roosting behaviour and/or other factors is likely to affect individual investment in cellular immunity over time and contribute to the species-specific responses of cellular immunity we observed (Patterson *et al.,* 2007; Allen *et al.,* 2008; Schneeberger *et al.,* 2013). The presence of other bacterial or protozoan infections may be responsible for neutrophilia and/or lymphopenia (Wyllie *et al.,* 2004; Liew and Kubes, 2019); however, we cannot confirm this without data on other infectious agents.

Our findings suggest different species of bats experience changes in cellular immune defenses over time, some of which may be linked to habitat loss and fragmentation from land conversion. However, we did not find evidence for these changes being associated with increased risk of pathogen infection for the pathogens quantified. Absence of significant changes in infection may be attributed to similar rates of pathogen exposure over time and/or low levels of *Bartonella* spp. and hemoplasmas in the blood. Additionally, the ability to feed on diverse prey (*D. rotundus*) or select early-successional habitats for preferred fruit (*S. parvidens*) may buffer these bats from stressors associated with habitat loss and fragmentation. Although the matrix beyond the LAR is being converted to rangeland overtime (Herrera *et al.,* 2018; Ingala *et al.,* 2019, Figure. 1), our study site remains relatively large and contains mature trees and caves for roosting. If prey availability, caves and mature forests persist in the LAR, bats that rely on these dietary and shelter niches may be able to persist without experiencing increased pathogen exposure from habitat reduction.

Although our findings suggest that our select bat species invested differently in immune defenses and that temporal patterns of immune investment varied among species, further research is needed to establish the role of dietary habits in shaping such trends. We selected one representative species per dietary habit for this analysis, but replication is needed to better establish the role of foraging ecology relative to bat taxonomy and other species traits. For example, roosting behaviour, body size, sociality, and preferred habitat type are other factors that may influence how land use changes affect bats and their immunity (Schneeberger *et al.,* 2013; Ávila-Gómez *et al.,* 2015). Comparisons of other frugivorous and insectivorous species, across periods of land conversion would be highly informative, given the rarity of sanguivory among bats. Similarly, our single included insectivore (*P. mesoamericanus*) belongs to the sister family (Mormoopidae) of the other two species (members of Phyllostomidae); inclusion of insectivorous or nectarivorous and/or omnivorous phyllostomids would facilitate more comprehensive comparisons within a clade.

Due to the important ecological roles played by bats in Neotropical ecosystems (e.g. seed dispersal, pollination), understanding how land conversion impacts bat immunity is critical to conservation of both species and ecosystem function. Frugivorous bats, such as *S. parvidens*, play essential roles in forest regeneration in Neotropical ecosystems (Mello *et al.,* 2008), while insectivorous bats, such as *P. mesoamericanus*, are important predators of insects (Alpízar *et al.,* 2019; Núñez *et al.,* 2019), including families that are considered agricultural pests (Ingala *et al.,* 2021). Likewise, the preservation of mature trees (the preferred roosting habitat of *S. parvidens*), is essential for the conservation of *S. parvidens* (Galindo-Gonzalez *et al.,* 2000; Evelyn and Stiles, 2003). When land conversion reduces access to mature trees, the physiological condition of these bats could suffer, and consequently, result in reduced immunocompetence. Similarly, *P. mesoamericanus* requires dense forest interiors to forage for insects, which makes them particularly vulnerable to the immunological stressors of major habitat loss and fragmentation (Alpízar *et al.,* 2019; Núñez *et al.,* 2019). In terms of conservation, while *S. parvidens* and *P. mesoamericanus* are of Least Concern status according to the IUCN (Solari, 2016; Solari, 2019), they are susceptible to extirpation in landscapes void of dense, mature forest. Thus, we suggest monitoring these species in areas experiencing increasing land conversion (Galindo-Gonzalez *et al.,* 2000; Evelyn and Stiles, 2003; Hernández-Canchola and León-Paniagua, 2020). Lastly, understanding how habitat modification impacts the immunity of *D. rotundus* is important for monitoring rabies virus transmission and control strategies throughout the Neotropics (Becker *et al.,* 2021*b*).

In sum, our study provides evidence that *D. rotundus*, *S. parvidens* and *P. mesoamericanus* invested differently in cellular immunity in ways that changed over time, suggesting that investment in immune defenses varies by species and dietary habit. Despite this, we found no evidence that *Bartonella* spp. or hemoplasma infection risk in these bats changed over time, although comparisons were limited by small sample sizes and the short time span of our study. Comparisons of infection prevalence of bats between habitat undergoing rapid land conversion and habitat remaining intact should be made to determine the role of habitat degradation in shaping infection risk. Studying the immunological effects of land use changes on Neotropical species is important for wildlife management, disease management and conservation. Continual monitoring of the bat species included in this study is recommended as their habitat becomes increasingly fragmented.

## Supplementary Material

Web_Material_coad102

## Data Availability

All 16S rRNA sequence data are available on GenBank through accession OQ308885–7, OQ308889, OQ308893, OQ308895–97, OQ308899–900, OQ308902–12, OQ308914–19, OQ308923–24, OQ308926–27, OQ533048, OQ546518–19, OQ546527–28, OQ546533, OQ546547, OQ546552 and OQ546555.
